# Adherence to Medical Treatment in Inflammatory Bowel Disease Patients from a Referral Center in Bahia-Brazil

**DOI:** 10.1155/2020/5269493

**Published:** 2020-09-26

**Authors:** Laíla D. Andrade, Fernanda A. Oliveira, Victor D. Mariano, Monique C. A. Santos, Fernanda A. Pereira, Cláudia I. N. dos Santos, Flora M. L. Fortes, Andrea M. Pimentel, Jaciane A. Mota, Neogélia P. Almeida, Valdiana C. Surlo, Bruno C. Silva, Mila P. Pacheco, Genoile O. Santana

**Affiliations:** ^1^Medical School, Centro Universitário UniFTC, Salvador, Bahia, Brazil; ^2^Department of Life Sciences, Universidade do Estado da Bahia, Salvador, Bahia, Brazil; ^3^Gastroenterology Unit, Hospital Geral Roberto Santos, Salvador, Bahia, Brazil; ^4^Gastroenterology Unit, Hospital da Bahia, Salvador, Bahia, Brazil

## Abstract

**Methods:**

Observational, analytical, and cross-sectional studies carried out from June/2017 to July/2018, with questionnaire application and medical record review at a referral center in inflammatory bowel diseases in Salvador, Bahia. The Morisky Green Levine Scale was applied to assess adherence. Mean, standard deviation, and frequency analyses were performed using the statistical package SPSS, and chi-square was used to evaluate the association between categorical variables and adherence degree to treatment. Significant associations were considered with *p* < 0.05.

**Results:**

302 patients with inflammatory bowel diseases were included. Nonadherence was highlighted in the sample. Most part of the study population was female, declared themselves to be mixed race, claimed to be from urban areas, and married. Nonadherence was more frequent than adherence in most sociodemographic variables of the present study. Nonadherence also stood out among the clinical variables, such as disease activity, drug side effect, and use of more than two additional medications. The association between all studied variables and adherence degree to treatment, considering the general sample, did not show statistical significance. When Crohn's disease and ulcerative colitis patients were evaluated separately, a statistically significant association between nonadherence and female patients with ulcerative colitis was observed.

**Conclusions:**

The high frequency of nonadherence was observed in the studied sample. Female gender was associated to nonadherence in the subpopulation with ulcerative colitis.

## 1. Introduction

Inflammatory bowel disease (IBD) encompasses a range of diseases that affect the gastrointestinal tract, among which the most prevalent are Crohn's disease (CD) and ulcerative colitis (UC). It consists of a chronic disorder with a rising global incidence [1] and results in significant morbidity, in addition to compromising the quality of life of patients [2], both due to the intestinal symptoms of the disease itself, as well as its complications, such as hospitalization rates, possible surgical interventions [3], and extraintestinal manifestations, which can affect one-third of patients at any stage of the disease [4].

In the IBD scenario, drug therapy is aimed at inducing remission, subsequent maintenance of this remission, and nutritional balance [5]. Conventionally, it acts in blocking different cells, receptors, and mediators participating in the inflammatory cascade [6]. The first line of treatment for colonic CD and mild to moderate UC is aminosalicylates. When there is no adequate clinical compensation with maximum doses of oral and topical salicylates, the addition of immunosuppressants is indicated, with azathioprine being the most used, which can initially be prescribed in conjunction with oral corticosteroids. In cases of insufficient response with immunosuppressants, immunobiological therapy, Janus kinase inhibitors, or surgery are usually the next treatment step [7].

The evaluation methods of adherence to pharmacotherapy can be conceptualized directly, which includes biochemical analysis for detection of the drug or metabolite in the plasma or urine, and indirectly, such as patient interviews with self-rated adherence tests or scales, patient diary, manual pill counting, monitoring dispensation record, perception of the doctor, or health professional [8, 9].

Despite more than five decades of research on the topic, the problem of nonadherence persists in the daily routine of health services [10]. There is an urge to verify the degree of medication adherence and understand its associated factors, to better adapt the health care team's conduct based on scientific evidence.

The present study is aimed at identifying the degree of adherence to drug therapy in patients with IBD followed up at a referral center in Bahia-Brazil, as well as to associate sociodemographic variables, disease activity, side effects, and polypharmacy with the degree of medication adherence.

## 2. Materials and Methods

Observational, analytical, and cross-sectional studies carried out at an IBD referral center of specialized care according to the State Program for High-Cost Medicine of the Bahia State Department of Health are used. From June 2017 to July 2018, structured interviews and analysis of medical records were performed to complete questionnaires of patients diagnosed with IBD, with clinical, histopathological, and endoscopic confirmation according to the European consensus of Crohn's and colitis criteria [11]. The recruitment was done during routine medical consultation at the outpatient IBD referral clinic. Individuals diagnosed with IBD aged over 18 years were included. Those who did not use any drug therapy for IBD and patients unable to answer the questionnaire were excluded from the study.

The questionnaire addressed items related to epidemiological data, including gender, age, ethnicity/color, origin, marital status, education; variables related to medication use for IBD, and other chronic diseases, considering use of more than two additional long-term medication for chronic diseases in addition to IBD; items related to disease activity through the Harvey-Bradshaw Index [12], which establishes the clinic progression of CD from the assessment of general well-being, occurrence of abdominal pain, number of liquid or soft stools in the previous day, presence of abdominal, mass and systemic complications; and Lichtiger score [13], which establishes the progression of the UC from the assessment of general well-being, occurrence of diarrhea, nocturnal diarrhea, visible blood in the stools, fecal incontinence, and abdominal pain; variables related to location and phenotype of the disease according to the Monteral classification for CD and UC [11]; and finally, items related to medication adherence by applying the treatment adherence test proposed by Morisky et al. [14], known as Morisky Green Levine Scale, an instrument that indirectly analyzes the degree of patient adherence to treatment, and consists of four questions: (1) Do you ever forget to take your medicine? (2) Are you careless at times about taking your medicine? (3) When you feel better, do you sometimes stop taking your medicine? (4) Sometimes if you feel worse when you take the medicine, do you stop taking it? This needs only one positive response to be considered nonadherent [5, 15].

The results were analyzed using the statistical package SPSS version 21.0; the mean, standard deviation, and frequency were calculated; and the chi-square test was applied to evaluate the association between categorical variables and the degree of adherence to treatment. Associations with *p* value less than 0.05 were considered statistically significant.

This study was conducted in compliance with the principles of the Declaration of Helsinki. The protocol was reviewed and approved by the Research Ethics Committee of the Roberto Santos General Hospital (64959317.9.0000.5038). Written informed consent was presented and signed by patients prior to the application of the questionnaires, as well as before reviewing medical records.

## 3. Results

This study included 302 patients, 116 with CD and 186 with UC. The majority of the sample was female, the average age of the individuals interviewed was 45.8 years (SD ± 15.05), more than half of the study population declared themselves mixed race, 80.5% of the individuals claimed to be from an urban area, 44.5% (*n* = 134) had completed high school, and most were married, as shown in Table 1.

According to the Montreal Classification, most individuals were between 17 and 40 years old at the time of diagnosis of CD. Most of the sample had colonic localization involvement, followed by those with ileocolonic presentation. Regarding the behavior of the disease, more than half of the interviewees presented nonstricturing nonpenetrating behavior, as shown in Table 2.

The Montreal Classification for UC demonstrated that the same number of individuals had left colitis and extensive colitis, and the minority had proctitis (Table 3).

Nonadherence was observed in 72.7% (218/302) of the sample. Therapeutic adherence was represented in 83 (27.3%) individuals. Among the 116 patients with CD, 68.1% were considered nonadherent. As for individuals with UC, 75.3% of the 186 patients were nonadherents. There was no statistical significance between the type of IBD and therapeutic adherence.

Analyzing IBD as a whole, the chi-square analysis performed between the degree of adherence and the sociodemographic variables did not show statistical significance. However, when analyzing UC and CD separately, the study found a *p* = 0.018 in the association between gender and adherence in UC patients (see Table 4).

When evaluating an association between medication nonadherence and disease activity, side effects, and use of more than two additional medication, no statistically significant difference was observed (Table 5).

## 4. Discussion

In an attempt to achieve the most drug efficacy, patients must follow the prescribed therapeutic regimens. There is no agreement on the cut-off point that defines proper adherence. Studies consider that rates above 80% of adherence are acceptable, while others consider rates above 95% to be an appropriate requirement to define therapeutic adherence. The context of IBD is not different; the level of nonadherence varies from 7% to 72% between studies [16]. This data varies according to the population studied, study location, and the method applied for adherence analysis, whether indirect or direct [8].

The Morisky Green Levine Scale is an indirect method for adherence analysis, with sensitivity of 81% and specificity of 44% [8]. It has the characteristic of overestimating nonadherence, due to the rigidity of its criteria to consider an adherent patient [5, 14, 17].

Due to its relapsing chronic character, with alternation between periods of remission and disease activity and the consequent need for complex therapeutic alternatives with medications of different pharmacological classes and long-term dosages, IBD is classified as a high probability condition for nonadherence [18] which is in agreement with 72.7% of nonadherence present in the current sample. Most studies that disagree with this percentage [19–21] use other methods of analysis for adherence degree, such as 8-item Morisky Medication Adherence Scale which has items addressing the circumstances of adherence behavior; this score presents conflicting data in the literature about its performance in IBD patients [8]. Another explanation could be the fact that many patients tend to overestimate their adherence when questioned by their physicians, thus decreasing specificity and increasing false positive results [20]. In the case of the current study, the data collection was performed by medical students in an environment outside the medical care room, which possibly contributed to the patient not feeling the need to overestimate their adherence to the doctor-oriented treatment.

In the other studies that used the Morisky Green Levine Scale to assess the degree of adherence in IBD patients [5, 15], the nonadherence behavior agrees with the present study as it stands out as more prevalent. Similar to the present study, a conducted research in Minas Gerais showed a 64% prevalence of nonadherence to drug treatment among patients with CD [15]. As well as in the paper carried out in Spain, the frequency of nonadherence was 72% of the individuals included [22]. The study by Sewitch et al. [17], one of the first to use the Morisky Green Levine Scale for IBD, revealed nonadherence in less than half of the sample (41.2%) of 153 IBD patients involved [17]. This disagreement may be related to the difference in sample size of the studies, considering that the sample corresponds to approximately half of the present study, as well as the population distribution difference, as the study by Sewitch et al. [17] has more than half (69.3%) of individuals with CD, which is almost opposite to the sample of the current study. As well as the location of the study, which was conducted in Canada, a country that has a higher human development index than Brazil, which, according to the United Nations Development Program, reflects a worse Brazilian social condition [15], which according to the World Health Organization is one of the factors involved in therapeutic adherence [23].

A study carried out in São Paulo, which also uses the Morisky Green Levine Scale, demonstrates 63.3% of nonadherence rate among the 30 patients with UC, similar to the sample in the current study; however, it shows 50% of nonadherence, among the 26 patients interviewed with CD [5]. It is possible to attribute this result to a small number of individuals included in the study, as well as the predominance of individuals with partners and a statistically significant association (*p* = 0.01) between higher adherence and stable marital status in the sample, considering that it has already been observed that nonadherence seems to be more likely in unmarried patients [16]. In the present study, the difference between marital status and treatment adherence was not statistically significant.

An Italian study involving 151 patients with IBD showed a 71.5% adherence rate, revealing a high prevalence of this behavior in relation to the other studies described [24]. The main difference when compared with the current study was the use of the Morisky Medication Adherence Scale-8 as an adherence assessment test, this being filled out by the patient anonymously, which has conflicting data when related to studies for IBD [8], in addition to the high cost for its application. The Italian study found no statistical significance in the association among gender, type of IBD, and disease activity with the behavior of adherence to therapy. The greatest impact factor for nonadherence consisted of complex therapeutic plans, with the use of more than one drug, and the greatest risk for nonadherence involves patients at young age (<30 years) [24].

Nonadherence was more frequent than adherence in most sociodemographic variables of the present study. In general, the sociodemographic profile of the patients included in the study was: female, self-declared mixed race, from the urban areas, married, and completed high school. When analyzing UC and CD separately, the study found statistical significance when correlating adherence with UC in female patients (*p* = 0.018). A meta-analysis [10] involving 569 studies on medication adherence in chronic diseases did not show a statistically significant difference between gender and nonadherence, although it signals that the prevalence of adherence is higher among female patients. Other studies [18–20, 22, 24, 25] that used indirect methods (application of questionnaires) to assess adherence in patients with IBD did not show statistical significance between nonadherence behavior and gender. In a systematic review that addresses risk factors associated with poor adherence in IBD, the results regarding gender were contradictory, showing that for all studies that report a significant association between any demographic variable and poor adherence, there were at least the same number of studies reporting no significant association between this variable and nonadherence [16].

However, our finding is correlated with study involving 376 IBD patients using mesalazine, in which a lower nonadherence rate was found in the female sample (*p* = 0.015) [26]. This finding was extended to the subpopulation with CD (*p* = 0.006), but not applicable to patients with UC (*p* = 0.6) [26]. It is also worth mentioning that our study presents more than two-thirds of the sample as female, which may have contributed to a greater emphasis on the nonadherence rate in this gender.

As in the present study, considering the general sample with IBD, the literature also points out that factors such as disease activity do not influence, in a statistically significant way, therapeutic adherence [19, 26]. However, such data differ from other studies that when evaluating the association between intentional nonadherence in patients with CD and low scores in the Harvey-Bradshaw Index showed statistical significance, indicating that individuals with lower disease activity would be less adherent [22]. This finding also can be seen in an Italian study [25] involving 485 patients with IBD, which shows a higher prevalence of nonadherence in patients in remission of the disease (*p* < 0.001). This study, however, used a 24-item self-administered questionnaire, in which the disease activity was measured through the patient's self-perception. Other studies point out that, in patients in remission from IBD, medication adherence decreases over time and is harder to maintain than in active disease [20]. This can be attributed to the lower intensity and number of symptoms in the long-term remission periods, symptoms that would act as a constant reminder of the importance of following the treatment properly.

The lack of statistical significance when associating medication adherence to use of more than two additional medication and side effects is in agreement with other studies [19, 26]. Many studies were not concerned with evaluating this association, making it impossible to compare results. However, there are reports that a complex therapy with many drugs not only for intestinal disease would represent an increased risk for nonadherence to immunomodulators [26].

Among the limitations of the study, the use of an indirect method to ascertain the degree of adherence, which was chosen because of its easy application, good acceptance by interviewees, and lower cost but has intrinsic limitations of a method that is based on the accuracy of the respondents' response. In studies comparing adherence rates by direct and indirect methods, it was observed that the vulnerability of indirect methods is associated with a higher probability of overestimation of adherence rather than nonadherence. The Morisky Green Levine Scale, in turn, despite being indirect, has the characteristic of favoring nonadherence results [5].

## 5. Conclusions

In conclusion, a high frequency of nonadherence was observed in the studied sample. No statistically significant association was observed among sociodemographic variables, use of more than two additional medication, side effects, and disease activity in relation to nonadherence to drug treatment, considering the general sample. Female gender is statistically significantly associated to nonadherence in the subpopulation with UC.

## Figures and Tables

**Table 1:** Sociodemographic characteristics in inflammatory bowel disease patients from Salvador/BA 2017/2018.

Gender	
Female	190 (62.9)
Male	112 (37.1)
Age (yr)	45.8 ± 15.05
Ethnicity/color-self-declaration	
Mixed race	158 (52.7)
Black	110 (36.7)
White	22 (7.3)
Asian	7 (2.3)
Not declared	3 (1.0)
Origin	
Urban area	243 (80.5)
Countryside	52 (17.2)
Not declared	7 (2.3)
Scholarity	
High school	134 (44.5)
Literate^b^	65 (21.6)
Primary school	56 (18.6)
University	42 (14.0)
Illiterate	3 (1.0)
Not declared	1 (0.3)
Marital status	
Married	135 (45.0)
Single	133 (44.3)
Divorced	20 (6.7)
Widower	12 (4.0)

Values are presented as mean ± SD or number (%). ^b^Only read and write, without complete supplementary education.

**Table 2:** Montreal classification in Crohn's disease patients from Salvador/BA 2017/2018.

Age at diagnosis	
A1: ≤16 years	5 (4.3)
A2: 17-40 years	82 (70.7)
A3: >40 years	29 (25.0)
Location	
L1: terminal ileum	21 (18.4)
L2: colon	49 (43.0)
L3: ileocolonic	44 (38.6)
L1+upper GI tract (L4)^a^	5 (4.4)
L2+L4	4 (3.5)
L3+L4	6 (5.3)
Behavior	
B1: nonstricturing/nonpenetrating	59 (53.2)
B2: stricturing	25 (22.5)
B3: penetrating	27 (24.3)
B1+perianal disease (p)^b^	24 (21.6)
B2+(p)	6 (5.4)
B3+(p)	14 (12.6)

Values are presented as number (%). ^a^Upper gastrointestinal tract modifier (L4) allows location coclassification L4 through L1 to L3. ^b^(p) modifier perianal disease, added to B1 to B3, if concomitant perianal disease.

**Table 3:** Montreal classification in patients with ulcerative colitis from Salvador/BA 2017/2018.

Proctitis	23 (12.6)
Left colitis	79 (43.7)
Extensive colitis	79 (43.7)

Values are presented as number (%).

**Table 4:** Association between adherence to treatment according to sociodemographic variables in inflammatory bowel disease patients from Salvador/BA 2017/2018.

	IBD nonadherence	UC nonadherence	CD nonadherence
Gender			
Female	144/190 (75.8)	97/120 (80.8)^a^	47/70 (67.1)
Male	74/112 (66.1)	43/66 (65.2)	32/46 (69.6)
Marital status			
Single	103/133 (77.4)	56/67 (83.6)	47/66 (71.2)
Married	95/135 (70.4)	69/95 (72.6)	26/40 (65.0)
Divorced	13/20 (65.0)	9/13 (69.2)	4/7 (57.1)
Widower	6/12 (50.0)	5/10 (50.0)	1/2 (50.0)
Scholarity			
High school	98/134 (73.1)	58/75 (77.3)	40/59 (67.8)
Literate^b^	42/65 (64,6)	33/48 (68.6)	9/17 (52.9)
Primary school	43/56 (76.8)	27/33 (81.8)	16/23 (69.6)
University	34/42 (81.0)	21/27 (77.8)	13/15 (86.7)
Illiterate	0/3 (0.0)	0/2 (0.0)	0/1 (0.0)
Not declared	1/1 (100.0)	0/0 (0.0)	1/1 (100.0)
Ethnicity/color-self-declaration			
Mixed race	111/158 (70.3)	74/100 (74.0)	37/58 (63.8)
Black	83/110 (75.5)	51/68 (75.0)	32/42 (76.2)
White	14/22 (63.6)	5/8 (62.5)	9/14 (64.3)
Asian	6/7 (85.7)	5/5 (100.0)	1/2 (50.0)
Not declared	3/3 (100.0)	3/3 (100.0)	0/0 (0.0)
Origin			
Urban area	183/243 (75.3)	117/151 (77.5)	66/92 (71.2)
Countryside	34/52 (65.4)	22/31 (71.0)	12/21 (57.1)

Values are presented as number/total (%). ^a^*p* < 0.05. ^b^Only read and write, without complete supplementary education. IBD: inflammatory bowel disease; UC: ulcerative colitis; CD: Crohn's disease.

**Table 5:** Association between treatment nonadherence and clinical variables in inflammatory bowel disease patients from Salvador/BA 2017/2018.

	IBD nonadherence^a^	UC nonadherence^a^	CD nonadherence^a^
Disease activity			
Remission	169/231 (73.2)	107/141 (75.9)	62/90 (68.9)
Active disease	50/68 (73.5)	33/43 (76.7)	17/25 (68.0)
Side effects			
With side effects	48/65 (73.8)	25/35 (71.4)	23/30 (76.7)
No side effects	130/188 (69.1)	86/116 (74.1)	44/72 (61.1)
More than two additional medication			
More than two additional medication	93/134 (69.4)	68/93 (73.1)	25/41 (60.9)
No additional medication	120/161 (74.5)	67/88 (76.1)	53/73 (72.6)

Values are presented as number/total (%). ^a^Not statistically significant. IBD: inflammatory bowel disease; UC: ulcerative colitis; CD: Crohn's disease.

## Data Availability

The data used to support the findings of this study are available from the corresponding author upon request.
